# The influence of leisure sports activities on social health in adults

**DOI:** 10.1186/s40064-016-3296-9

**Published:** 2016-09-22

**Authors:** Bădicu Georgian, Balint Lorand

**Affiliations:** Department of Physical Education and Special Motility, Faculty of Physical Education and Mountain Sports, Transilvania University of Brasov, No. 1 Colina Universităţii Street, 500068 Brasov, Romania

**Keywords:** Activities related to motility, Social relations, Sports, Questionnaire

## Abstract

**Objective:**

The objective of this article is to present a methodology based on a questionnaire focused on social health, our study aims at highlighting, in a declarative and comparative manner, the development of social relationships between individuals, more or less for both adults who perform leisure sports activities and for those who do not practice.

**Methods:**

The study was conducted between October 2012, March 2013 on a sample of 500 adults, who responded to a survey questionnaire, of whom 318 individuals perform leisure sports activities and 182 do not perform this kind of activities. We mention that the age range of our subjects undergoing our research is 25–49 years.

**Results:**

In statements, from the pool of persons who practice sports activities, the age categories with a high percentage of people enjoying a good health, are: 25–29 years (88.6 %) and 45–49 years (83.3 %). For the other age categories, a decreasing percentage of persons with a good social health is noted, with aging. On the persons who do not practice leisure sports activities we note instead, an increasing percentage of the persons with an unsatisfactory social health, from 43.3 up to 76 %, that comes with aging.

**Conclusions:**

The results suggest that constant and continuous practice of various physical exercises represents a significant factor, which leads to the improvement of social health.

## Background

A good health is an essential element of human wellbeing, which is a value in itself (Alber and Köhler 2004).

Health is a fundamental resource for individuals, communities and societies overall. For an individual, enjoying a good health is crucial. At the same time, a generally good level of health of the population is essential to the economic growth and social development.

OMS defines health not only as a simple absence of disease and infirmity, but also as a general “wellbeing”: physical, mental, social (World Health Organization 2012).

Health can be measured objectively (e.g.: the collection and analysis of some biological and clinical parameters, encompassed in a research/evaluation sheet, for each individual of a population), as well as subjectively. The most frequent method of subjective measurement of health is self-evaluation. Satisfaction questionnaires and/or evaluation questionnaires of one’s health are quite frequently used, usually together with an objective method of evaluation of health (Vladescu et al. 2010). Maintaining an optimal physical health is rather a delicate matter nowadays, which each society should acknowledge and be responsible for, in order to cope with the daily demands. Through the level of effort, man can diminish or increase his physical capacity and strength.

The ideal way to come in contact with other peers, to establish new relationships, to consume energy, is sport, as leisure activity, which leads to relaxation etc., but it can also lead to another aesthetic sense (Brîndescu 2010).

In terms of sport, one can state that social health plays or should play an important role, in the sense that practicing physical activities lead to the development of social relationships between individuals. It is well known how enjoyable are aerobic exercises when performed together with colleagues, group jogging, rides on bicycles and ski, sports games etc.

Sports activities, performed in group a stimulating and refreshing, and develop verbal and non verbal communication. Thus, this type of social health translates into a socialization and satisfaction increase (Epuran 2011).

Included in the syllabuses, massively present in mass-media and assiduously performed by some, sport is a contemporary phenomenon, that cannot be ignored, because more and more people are aware that performing physical activities ensures a high quality of life. A society that promotes sport and understands its value in life, is a society that ensures a solid component for social wellbeing and optimism (Rusu 2008).

In terms of social health (Esterson and Finke 1994) present the physical and mental, as well as the social benefits of sport, of which the most important are: playing a sport or physical leisure involve, most of the time, the enrollment in different organized sports activities; we meet persons who have common interests and we make new friends; we build a team work spirit and develop a sense of cooperation, that is also useful in extra sportive activity.

It is believed that social health represents the way a person behaves with others and the way others respond to this behavior, the way a person behaves towards the social institutions, and social rules. Based on the interaction between individual and his environment, social health can, in fact, be interpreted in different ways. For example, within the “Health inquiry”, in Belgium, in 2004, social health translates, particulary, into social support terms (the availability of others persons an individual trusts, that he can count on and make him feel appreciated and respected as a person).

Social health refers to one’s capacity of achieving his lifetime purpose, such as being a son or daughter, a parent, a husband, a friend or a citizen, in an effective and comfortable way, without disturbing the social and secure ecological climate of others. Each of this purposes comes with different responsibilities and risks. All purposes require an effective communication, the “give and receive” kind, since healthy relations are never accomplished in an unilateral way. The fulfillment of human needs for love, intimacy, collective belonging, represent an important factor in achieving social health. The persons who are deprived of these needs may develop behaviors that can threaten their health and wellbeing, and thus fall under the incidence of anomy, deviance and social pathology (Rusu 2008).

As for the social wellbeing, it means: an optimal habitat, proper nutrition, adequate socialization (acquaintances, school, friends, family). Currently, many health problems are caused by socio-economic factors, that, however, can be changed through a beneficial collective activity and work. The purpose of health education is to make people understand how their behavior and environment may affect their health. Health education has no age boundaries. Its purpose is to naturally promote “human wellbeing” and to offer practical means of disease prevention, through proper nutrition and a healthy lifestyle, which enables modern man to successfully get over stressful situations, which can lead to a potential bodily offbalancing (European Union Agencies 2012).

Different research programmes have shown a significant link between subjective health and each of the social health (wellbeing) indicators, whatever these may be. People who assess their health as poor have a greater tendency of being dissatisfied with their social contacts, have little contact with their entourage, have a limited social network and declare poor social and functional support (Enquête de Santé–Belgique 2001). The same study shows that the deficiency of social contacts (once a month, once a year, never) is reported by 8 % of the population of over the age of 35 (above the average of population); people over the age of 75 have a limited social network. Interestingly enough, this situation is also encountered at the age 15–24.

## Methods

The study was conducted between October 2012, March 2013 on a sample of 500 adults, of whom 318 individuals perform leisure sports activities and 182 do not perform this kind of activities. We mention that the age range of our subjects undergoing our research, is 25–49 years. The subjects are distributed as follows: aged between 25 and 29 y/o N = 65 (25 males; 40 females); aged between 30 and 34 y/o, N = 120 subjects (55 males and 65 females); for those aged between 35 and 39 y/o, we have N = 65 (males 39; females 26), subjects aged between 40 and 44 y/o, N = 175 (70 males and 105 females); 45–49 y/o, N = 75 (40 males and 35 females).

In this research, we have applied a questionnaire, focused on the main component of the quality of life–health, and on the subcomponent, social health. The questionnaire comprised 20 questions, with values of result interpretation between 1 and 60 points. The margins for the questionnaires they were: a-1 b-2 c-3 (points) and the final results for the 20 items are: from 1 to 20 “not satisfactory social health”; from 21 to 40 “average social health/satisfactory social health”; from 41 to 60 “good social health”. Every participant has checked one variant response.

This questionnaire was applied both at the beginning or at the end of the process of performing different leisure sports activities in Brasov town, and on the street, for all categories of adults, from the target group.

The questionnaire was administered to adult subjects who declared they performed leisure sports activities rather constantly (participants to various sports activities: cross, hiking, jogging, mountain running, running through parks, sports games, fitness, cycling, swimming, ski etc.), to persons to mentioned they did not perform sports activities (interviewed on the street), as well as to those who took part in different collective activities, organized by regional/local institutions, sports associations, non profit organizations etc. This questionnaire contained closed, open and anticipated answers.

After collecting and processing the results of the questionnaire, we used the SPSS software to calculate the alpha Cronbach index, the average, the standard deviation and other statistical analysis.

## Results

### Interpretation of data collected from the questionnaire that was applied

#### Validation of the questionnaire focused on social health (SPSS 20 2012)

For the social health questionnaire, Cronbach’s Alpha coefficient of validity scale with 20 items, has the value 0.790, which proves a good level of fidelity (Table 1). Also, we calculated the average and the standard deviation for the 500 interviewed subjects (Table 2).

**Table 1:** Degree of internal consistency of the questionnaire centered on social health

Cronbach’s alpha	No. of items
0.790	20

**Table 2:** Statistics related to the items of the social health questionnaire

	Average	SD	N
Social health represents?	2.23	0.763	500
Social health develops?	2.30	0.731	500
Considering all the things that will occupy your time during the week, how much time do you spend doing things that you love and that you enjoy?	2.27	0.739	500
Do you adapt fast to a new group of people?	2.21	0.785	500
Do you have close friends?	2.17	0.775	500
How is your relationships with your friends?	2.15	0.771	500
Do you feel distant in relation to your colleagues and you seek to avoid them?	2.20	0.765	500
Are you happy with your family life?	2.15	0.766	500
Do you meet often your parents, relatives or friends?	2.22	0.755	500
How good are your relationships with your family members?	2.22	0.772	500
How would you describe your social personality?	2.31	0.772	500
Would you mind if you had to stay isolated for a while, without communicating with anyone?	2.18	0.720	500
What is your income?	2.17	0.767	500
How much satisfaction does your current job bring you?	2.21	0.761	500
Are your work conditions satisfying?	2.19	0.784	500
Do you often feel under pressure at the workplace?	2.21	0.770	500
Do the works tasks assigned to you by your boss or his attitude distress you?	2.22	0.750	500
How much time do you spend, weekly, outside working hours, with your colleagues?	2.19	0.743	500
How would you describe your relationship with your work colleagues?	2.22	0.755	500
Does your work present less interest to you and do you feel your future is without any outlook?	2.21	0.761	500

### Social health, depending on the pool of subjects, in adults who practice leisure sports activities

By analyzing the pool of persons who practice leisure sports activities, we found that the average result for social health is 53.31, at N = 318 (SPSS 20 2012).

Variable values deviate from the average value, plus/minus, by 8.36.

Modular value shows us that value 59 is the most encountered on the interviewed subjects.

**Fig. 1:**
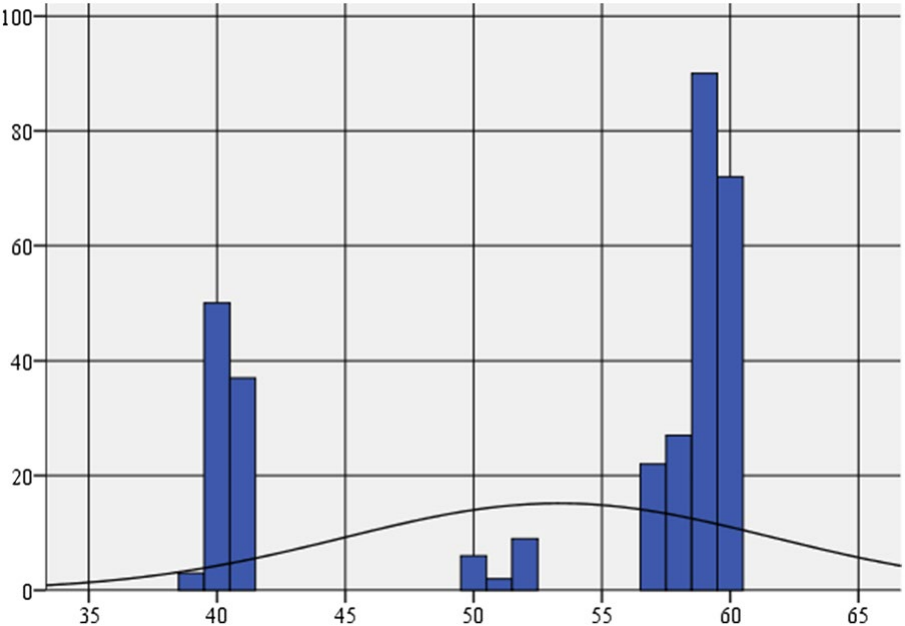
Histogram of the frequency distribution of social health on adults who practice leisure sports activities

 Kurtosis, of −1.191, indicates a platykurtic distribution. Asymmetry coefficient equal to −0.835 shows a negative asymmetric curve, tilted to the right, and multiple values to the left (Table 3; Fig. 1).

**Table 3:** Descriptive statistics on social health for the pool of persons who practice leisure sports activities

Social health status	
N	
Valid answers	318
Elliptical answers	0
Average	53.31
Median line	59.00
Module (modular value)	59
Standard deviation	8.369
Skewness asymmetry coefficient	−0.835
Kurtosis coefficient	−1.191
Minimum	39
Maximum	60

Practicing leisure sports activities = yes

Kolmogorov–Smirnov test, through shows us that the variable that was analyzed its result, is not distributed normally (Table 4).

**Table 4:** Testing the normality of scores distribution in terms of social health on persons who practice leisure sports activities

	Kolmogorov–Smirnov			Shapiro–Wilk		
	Value	Degrees of freedom df	Signification limit p	Value	Degrees of freedom df	Signification limit p
Social health status	0.334	318	0.000	−688	318	0.000

Practicing leisure sports activities = yes

**Fig. 2:**
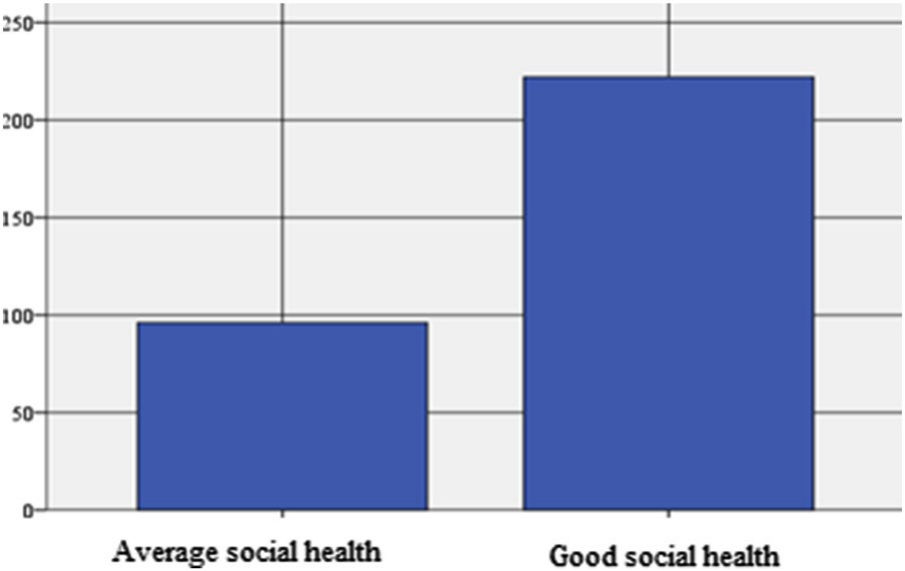
Distribution of the social health index on the persons who practice leisure sports activities

We mention that 69.8 % (N = 222) of the participants in leisure sports activities (N = 318) have a *good social health* (Table 5; Fig. 2). Out of the participants, 105 (70.5 %) of them are of male gender (aged between: 25 and 29 y/o, n = 12, i.e. 11.42 %; aged between 30 and 34 y/o, n = 25, i.e. 23.80 %; for the age range 35–39 y/o, n = 12, i.e. 11.42 %; 40–44 y/o, n = 37, i.e. 35.23 % and 45–49 y/o, n = 19, i.e. 18.1 %) and 117 (69.2 %) of them are of female gender. For the female gender, (N = 117), *with a good social health*, the distribution on age categories is as it follows: 25–29 y/o, n = 19, i.e. 16.23 %; 30–34 y/o, n = 33, i.e. 28.20 %, 35–39 y/o, n = 13, i.e. 11.11 %; 40–44 y/o, n = 36, i.e. 30.76 % and 45–49 y/o, n = 16, i.e. 13.67 %).

**Table 5:** Distribution of the social health index on the pool of persons who practice leisure sports activities

	Frequency	Percentage	Percentage of the valid answers	Cumulated percentage
Valid answers				
Average social health status (satisfactory)	96	30.2	30.2	30.2
Good social health status	222	69.8	69.8	100.0
Total	318	100.0	100.0	

Practicing leisure sports activities = yes

The subjects (N = 96) who obtained a score that corresponds to a *satisfactory* rating, following the declarations from the questionnaire (for the self-perception of the level of social health), represent a percentage of 30.2 out of the subjects who undergo sports activities, namely N = 318 (Table 5; Fig. 2) and 44 of them are males (29.5 %) and 52 females (30.8 %). As far as the male gender is concerned, the distribution on age categories is as follows: 25–29 y/o, n = 2, i.e. 4.54 %; 30–34 y/o. n = 6, i.e. 13.63 %; 35–39 y/o, n = 17, i.e. 38.63 %; 40–44 y/o, n = 10, i.e. 22.72 % and 45–49 y/o, n = 9, i.e. 20.45 %). For the female gender, N = 25, the subjects who stated they had *a satisfactory social health,* the distribution on age categories is as follows: 25–29 y/o, n = 4, (7.7 %); 30–34 y/o, n = 9 (17.3 %); 35–39 y/o, n = 13 (25 %); 40–44 y/o, n = 12, (23.07 %) and 45–49 y/o, n = 14 (26.92 %).

### Social health, depending on the pool of subjects, in adults who do not practice leisure sports activities

The average score in terms of social health on persons who *do not practice leisure sports activities*, (N = 182) is around 29.55, the most common value being 21 (SPSS 20 2012).

By analyzing the values distribution, it is noticed that theses values deviate from the average, plus or minus, by 9.47.

**Fig. 3:**
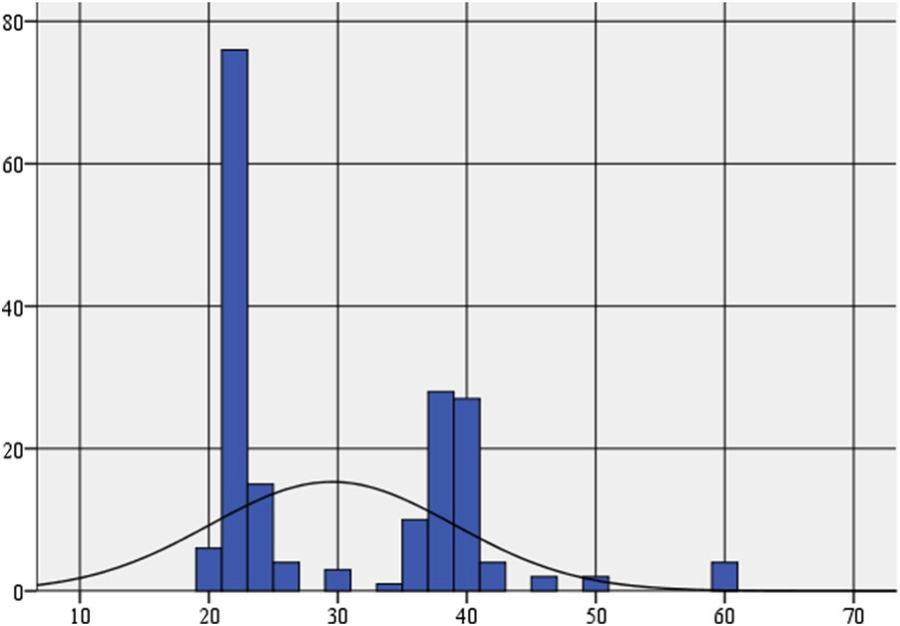
Histogram of the frequency distribution of social health on adults who do not practice leisure sports activities

The asymmetry coefficient (0.802) indicates a distribution tilted to the left, with multiple extreme values tilted to the right. The Kurtosis indicator (Kurtosis = −0.024) shows us a very slightly platykurtic distribution, very close to a normal distribution, with multiple values, dispersed on a larger range around the average (Table 6; Fig. 3).

**Table 6:** Descriptive statistics on social health for the pool of persons who do not practice leisure sports activities

Social health status	
N	
Valid answers	182
Elliptical answers	0
Average	29.55
Median line	23.00
Module (modular value)	21
Standard deviation	9.477
Skewness asymmetry coefficient	0.802
Kurtosis coefficient	−0.024
Minimum	20
Maximum	59

Practicing leisure sports activities = no

The variable is not distributed normally (Table 7).

**Table 7:** Testing the normality of scores distribution in terms of social health on persons who do not practice leisure sports activities

	Kolmogorov–Smirnov			Shapiro–Wilk		
	Value	Degrees of freedom df	Signification limit p	Value	Degrees of freedom df	Signification limit p
Social health status	0.283	182	0.000	0.799	182	0.000

Practicing leisure sports activities = no

**Fig. 4:**
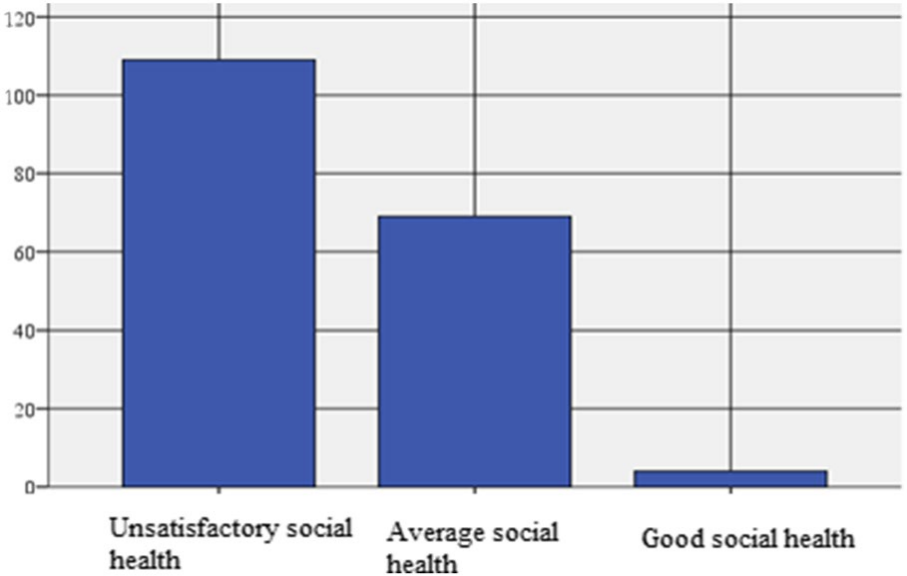
Distribution of the social health index on the adults who do not practice leisure sports activities

Of the 182 persons whose level of social health was assessed and who do not perform sports activities, the majority (59.9 %) have *an unsatisfactory social health*, 37.9 % have *an average social health*, whereas only 2.2 % have *a good social health* (Table 8; Fig. 4).

**Table 8:** Distribution of the social health index on the persons who do not practice leisure sports activities

	Frequency	Percentage	Percentage of the valid answers	Cumulated percentage
Valid answers				
Unsatisfactory social health status	109	59.9	59.9	59.9
Average social health status (satisfactory)	69	37.9	37.9	97.8
Good social health status	4	2.2	2.2	100.0
Total	182	100.0	100.0	

Practicing leisure sports activities = no

We note that 59.9 % (N = 109) of the total persons who do not practice leisure sports activities (N = 182) have *an unsatisfactory social health*. Out of them, 52 (64.2 %) are males (aged between: 25–29 y/o, n = 10, i.e. 19.23 %; 30–34 y/o, n = 11, i.e. 21.15 %; 35–39 y/o, n = 13, i.e. 25 %; 40–44 y/o, n = 10, i.e. 19.23 % and 45–49 y/o, n = 8, i.e. 15.38 % and 57 females. For the female gender, 56.4 % have *an unsatisfactory social health,* with the following distribution on age categories: 25–29 y/o, n = 9, i.e. 15.78 %; 30–34 y/o, n = 13, i.e. 22.80 %; 35–39 y/o, n = 18, i.e. 31.57 %; 40–44 y/o, n = 9, i.e. 15.78 % and 45–49 y/o, n = 8, i.e. 14.03 %.

The subjects (N = 69) who, following the declarations from the questionnaire, obtained a score corresponding to a *satisfactory rating* (for the self-perception of the level of social health), represent a percentage of 37.9 out of the subjects who do no practice sports activities, namely N = 182 (Table 8; Fig. 4) and 29 of them are males (35.8 %) and 40 females (39.6 %). As for the male gender, the distribution on age categories is as follows: 25–29 y/o, n = 4, i.e. 13.79 %; 30–34 y/o, n = 6, i.e. 20.68 %; 35–39 y/o, n = 8, i.e. 27.58 %; 40–44 y/o, n = 5, i.e. 17.24 % and 45–49 y/o, n = 6, i.e. 20.68 %. For the female gender, N = 40, the subjects who declared *a satisfactory social health,* are distributed on age categories as follows: 25–29 y/o, n = 10 (25 %); 30–34 y/o, n = 12 (30 %); 35–39 y/o, n = 10 (25 %); 40–44 y/o, n = 4 (10 %) and 45–49 y/o, n = 4 (10 %).

In the case of the subjects (N = 4) who, following the declarations from the questionnaire, came under *a good social health*, we state that these represent a percentage of 2.2 of the subjects who do not perform leisure sports activities, namely N = 182 (Table 8; Fig. 4) and none of them are males (0 %) and 4 females (4 %).

In the case of male gender, there is no distribution on age categories and for the female gender, N = 4, the subjects who declared *a good social health*, are distributed as follows: 25–29 y/o, n = 0 (0 %); 30–34 y/o, n = 0 (0 %); 35–39 y/o, n = 2 (50 %); 40–44 y/o, n = 1 (25 %) and 45–49 y/o, n = 1 (25 %).

## Discussion

The main evaluative component of the quality of life is health, and health depends on the level of physical, mental and social health, these three factors influencing each other.

Practicing physical exercises is the way most within our grasps to really influence in a beneficial way the health condition. Thus, effects are highlighted in regards to: body and segments harmony, maintaining the optimal parameters of the body vital functions, ensuring the necessary physical support for achieving the professional and social tasks, the development of a movement range necessary for professional, social and leisure activities, the development of some qualities in terms of personality (perseverance, self-government, self-control, sociability), the changement of mentality related to effort and practicing physical exercises as a part of lifestyle, obtaining/maintaining a physical condition, maintaining the health condition at an optimal level, inducing a general wellbeing and increasing the satisfaction level (Leonte 2012).

In terms of sport, Epuran (2011) says that, social health plays or should play an important role, in the sense that practicing physical activities lead to the development of social relationships between individuals. It is well known how enjoyable are aerobic exercises when performed together with colleagues, group jogging, rides on bicycles and ski, sports games etc. Sports activities, performed in group are stimulating and refreshing, and develop verbal and non verbal communication. Thus, this type of social health translates into a socialization and satisfaction increase.

There is a trend of growth of social dissatisfaction manifested with aging on both categories of subjects (the ones who practice/do not practice leisure sport). This trend is found to be more pronounced in passive persons/not participating in organized sports activities.

## Conclusions

Health is a fundamental resource for individuals, communities and societies overall. For individuals, enjoying a good health is essentially important. Also, a good health of the population overall, is essential to the economic growth and the development of the society (Alber and Köhler 2004).

An improved physical health/maintained through various exercises, represents a determinant factor for mental health, as well as for social health, and for the quality of life of each individual by default.

The research data show that the persons who practice leisure sports activities on a regular basis, have a higher social health index, unlike the persons who do not participate in this type of dynamic activities.

In statements, from the pool of persons who practice sports activities, the age categories with a high percentage of people enjoying a good health, are: 25–29 years (88.6 %) and 45–49 years (83.3 %). For the other age categories, a decreasing percentage of persons with a good social health is noted, with aging.

On the persons who do not practice leisure sports activities we note instead, an increasing percentage of the persons with an unsatisfactory social health, from 43.3 up to 76 %, that comes with aging.

## References

[CR1] Alber J, Köhler U (2004) Health and care in an enlarged Europe. Office for Official Publications of the European Commission, Luxembourg, p 35; (electronic version)

[CR3] Brîndescu S (2010) Beneficiile practicării activităţilor de timp liber. Revista Marathon, vol 2. Timişioara, pp 1–7

[CR2] Enquête de Santé – Belgique (2001) Institut Scientifique de la Santé Publique

[CR4] Epuran M (2011) Motricitate şi psihism în activităţile corporale, vol 1. Editura FEST, Bucureşti, pp 55–154

[CR5] Esterson P, Finke R (1994) Fitness: a way of life. Physical therapy association. APTA, p 14

[CR6] European Union Agencies (2012) Available April 10, 2012 from http://europa.eu/agencies

[CR7] Leonte N (2012) Bunăstarea fizică—dimensiune a calităţii vieţii. Revista Marathon, vol IV. Nr. 1, p 52

[CR8] Rusu F (2008) Note de curs fitness. Mutiplicat UBB, Cluj-Napoca, p 101

[CR9] SPSS 20 (Statistical Package for the Social Sciences) (2012) Available April 20, 2012 from http://analize-statistice.eu/

[CR10] Vlădescu C, Ciutan M, Mihăilă V (2010) Rolul măsurării mortalităţii evitabile în aprecierea stării de sănătate a populaţiei. Starea de sănătate a populaţiei—management în sănătate. XIV/3/2010, pp 5–11

[CR11] World Health Organization (2012) Available June 10, 2012 from http://www.who.int/about/definition/en/print.htm

